# SARS-CoV-2 transmission dynamics in Mozambique and Zimbabwe during the first 3 years of the pandemic

**DOI:** 10.1098/rsos.241275

**Published:** 2025-01-22

**Authors:** Roselyn F. Kaondera-Shava, Marta Galanti, Matteo Perini, Jiyeon Suh, Shannon M. Farley, Sergio Chicumbe, Ilesh Jani, Annette Cassy, Ivalda Macicame, Naisa Manafe, Wafaa El-Sadr, Jeffrey Shaman

**Affiliations:** ^1^ Department of Environmental Health Sciences, Mailman School of Public Health, Columbia University, New York, NY 10032, USA; ^2^ Department of Population and Family Health, Mailman School of Public Health, Columbia University, New York, NY 10032, USA; ^3^ Instituto Nacional de Saúde (INS) EN1, Bairro da Vila-Parcela n 3943, Distrito de Marracuene, Maputo, C.P. 264, Mozambique; ^4^ Department of Epidemiology, Mailman School of Public Health, Columbia University, New York, NY 10032, USA; ^5^ Columbia Climate School, Columbia University, New York, NY 10025, USA

**Keywords:** SARS-CoV-2, COVID-19, dynamic model, Bayesian inference, transmission rate, ascertainment rate

## Abstract

The 2019 emergence of severe acute respiratory syndrome coronavirus 2 (SARS-CoV-2) and its rapid spread created a public health emergency of international concern. However, the impact of the pandemic in Sub-Saharan Africa, as documented in cases, hospitalizations and deaths, appears far lower than in the Americas, Europe and Asia. Characterization of the transmission dynamics is critical for understanding how SARS-CoV-2 spreads and the true scale of the pandemic. Here, to better understand SARS-CoV-2 transmission dynamics in two southern African countries, Mozambique and Zimbabwe, we developed a dynamic model-Bayesian inference system to estimate key epidemiological parameters, namely the transmission and ascertainment rates. Total infection burdens (reported and unreported) during the first 3 years of the pandemic were reconstructed using a model-inference approach. Transmission rates rose with each successive wave, which aligns with observations in other continents. Ascertainment rates were found to be low and consistent with other African countries. Overall, the estimated disease burden was higher than the documented cases, indicating a need for improved reporting and surveillance. These findings aid understanding of COVID-19 disease and respiratory virus transmission dynamics in two African countries little investigated to date and can help guide future public health planning and control strategies.

## 1. Introduction

Following its emergence in China in late December 2019, severe acute respiratory syndrome coronavirus 2 (SARS-CoV-2), the virus that causes coronavirus disease 2019 (COVID-19), spread globally and produced an unprecedented public health crisis [1,2]. The World Health Organization (WHO) declared the COVID-19 outbreak a pandemic on 11th March 2020 [3]. In less than 3 years, in various parts of the world, including two southern African countries, i.e. Mozambique and Zimbabwe, SARS-CoV-2 triggered multiple waves of new infections, often driven by new variants of concern (VOC) [4].

The COVID-19 pandemic put enormous pressure on public health security; to mitigate COVID-19 spread, most African countries imposed travel restrictions or banned international travel and implemented curfews, lockdowns and other social distancing interventions beginning in March and April 2020 [5]. The initial cases of COVID-19 in Mozambique and Zimbabwe were reported on 22nd March and 20th March 2020 [6,7], respectively, and the pandemic subsequently spread to all provinces in both countries. By December 2022, three waves of infection, i.e. Alpha, Delta and Omicron (BA.1), had occurred.

Many SARS-CoV-2 infections are unreported; hence, confirmed case counts do not fully reflect epidemic dynamics. Understanding the level of ascertainment and the impact of undocumented cases on transmission is crucial to plan and implement control strategies. Models coupled with Bayesian inference methods have been used to investigate the role of undocumented infections in China [8], as well as for various regions (countries and US states) [9], 54 African countries [10] and 3 North African countries: Algeria, Egypt and Morocco [11]. Russel *et al*. [12] fitted a Bayesian Gaussian process model to estimate under-ascertainment in 210 countries and territories.

Characterization of transmission dynamics is critical for understanding how SARS-CoV-2 spread and the full extent of its impact on local populations. Studies have used statistical and modelling techniques to estimate key parameters like the reproductive number, ascertainment rates and total infections [13–15]. In addition, a model-inference approach was used to estimate the background population characteristics, such as population susceptibility, for South Africa [16], the United States at county resolution [17] and India [18].

In spite of this vast body of literature, characterization of SARS-CoV-2 transmission dynamics (or any respiratory virus) in the Southern African region remains mostly lacking, except for South Africa [16,19–21]. An autoregressive integrated moving average (ARIMA) model was utilized to forecast the trend of the disease in four African countries that reported the most cases: South Africa, Egypt, Nigeria and Ghana [22]. Few studies included Mozambique and Zimbabwe: Shoko & Juho [23] utilized ARIMA models to model and forecast the spread of the disease in Southern Africa Development Community (SADC) member states, which included both Mozambique and Zimbabwe. Han *et al*. [10] estimated the key epidemiological parameters for all 54 African countries (including Mozambique and Zimbabwe) using a mathematical model. Shoko *et al*. [24] used support vector regression for short-term forecasting of the disease in Zimbabwe. However, the reported COVID-19 impact (cases, hospitalizations and deaths) in Africa has likely underestimated the actual extent of infection and thus the transmission dynamics. Few studies have been published on COVID-19 in Mozambique and Zimbabwe; as a consequence, the investigation of key epidemiological characteristics over time and by VOC wave is needed.

Here, we utilize a model-inference framework to study COVID-19 disease dynamics in Mozambique and Zimbabwe during the first 3 years of the pandemic for three VOCs: Alpha, Delta and Omicron (BA.1). The dynamic model was coupled with a Bayesian inference method and province-specific case data. We accounted for undocumented cases to estimate key epidemiologic parameters, namely the transmission rate and the ascertainment rate at the end of each variant outbreak. The findings help advance understanding of the dynamics of SARS-CoV-2 and give information on the transmission dynamics of respiratory viruses in two African nations that have received little attention to date.

## 2. Methods

### 2.1. Epidemic transmission model

To describe the prevalent characteristics of COVID-19 in each province, i, for Mozambique and Zimbabwe, we used a susceptible–exposed–infectious–recovered (SEIR) compartmental model, similar to ones used in recent studies on COVID-19 [8,25–27]. The infected compartment was split into two: (i) infected reported ($I_{i}^{r}$) individuals who have tested positive and (ii) infected unreported ($I_{i}^{u}$) individuals who are infected with the virus but have not been tested and thus not reported. Unreported infections are not assumed to be asymptomatic, though a portion of this group is; however, it is a prior that unreported infections are on average less infectious than reported cases due to lower viral loads [28]. The susceptible population, $S_{i}$, is not yet infected; the exposed population, $E_{i}$, has been infected with the virus but does not yet transmit the virus. There is no reinfection of the recovered population, $R_{i}$ and $N_{i}$ is the total population.

**Figure 1.**
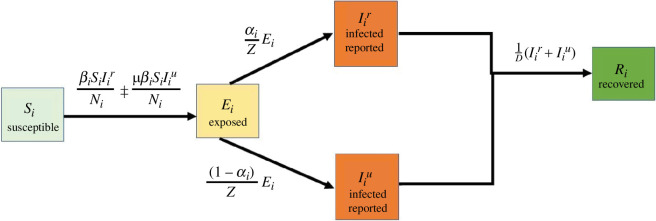
Schematic representation of simulated disease dynamics. The solid arrows demonstrate the movement from one disease state to another.

The model (figure 1) was coupled with data assimilation and case data to estimate the transmission rate, $\beta_{i}$, and the ascertainment rate, $\alpha_{i}$, at the end of each variant wave outbreak (Alpha, Delta and Omicron (BA.1)). We fitted the model to province-specific case data (i.e. number of new reported cases) as reported by the Mozambique Ministry of Health-SIGILIA and DISA [29] and the Zimbabwe COVID-19 hub [30] for the period between March 2020 and December 2022. Due to missing Zimbabwe COVID-19 provincial data, we only estimated parameters for the Omicron (BA.1) variant wave in Zimbabwe. The other parameters, i.e. average latency period, Z, average duration of infection, D, and reduction of infection rate for unreported infected individuals, μ, were kept constant (see electronic supplementary material, table S3). We also maintained a constant human population for the study period with no births or deaths.

#### 2.1.1. Model assumptions

The model is based on the following assumptions:

- there is no human movement between the provinces;
- individuals who are infected (reported and unreported) can transmit the virus to those susceptible;
- there is no reinfection during each variant wave period;
- population at steady state (births replacing deaths during the study period).

#### 2.1.2. Disease dynamics

The system of ordinary differential equations was adapted from Li *et al*. [8]. Figure 1 presents a schematic of model transmission dynamics, and a description of the model variables and parameters are shown in electronic supplementary material, table S1.

Model disease dynamics are described by a system of ordinary differential equations, electronic supplementary material, equations (S1)–(S4), which are subject to the non-negative initial conditions and satisfy the conservation of the population, i.e. $N_{i}=S_{i}+E_{i}+I_{i}^{r}+I_{i}^{u}+R_{i}$ for each province i. The basic reproductive number at time t is $Rt_{i}=\alpha_{i}\beta_{i}D+(1-\alpha_{i})\mu \beta_{i}D$, obtained through the next-generation approach (see [8] for a detailed explanation).

In our analysis, the SEIR model was integrated stochastically forward in time. A Latin hypercube sampling (LHS) technique was used to select a random set of initial variables and parameter combinations from prior ranges, which are assumed to be uniformly distributed. The initial priors for the variables and parameters are given in electronic supplementary material, table S3. A fourth-order Runge–Kutta (RK4) scheme was used for stochastic integration; in particular, to introduce stochasticity to the right-hand side of the system, a random sample from a Poisson distribution was used for each step of the RK4 scheme to determine each term.

For both countries, all provinces were run simultaneously as a metapopulation model without representation of human movement between provinces, due to the absence of robust mobility data. Attempted simulations using a gravity model to estimate interprovincial movement did not yield credible results (described in electronic supplementary material, §S1.6). As a consequence, we assumed disease transmission dynamics to be principally determined by local within-province processes.

#### 2.1.3. Piecewise simulation and inference

The metapopulation model form was coupled with the ensemble adjustment Kalman filter (EAKF), a Bayesian sequential ensemble filtering method and province-specific COVID-19 case data. Piecewise simulation and parameter estimation were performed separately for each variant wave: Alpha, Delta and Omicron (BA.1). The data periods for this piecewise inference are shown in electronic supplementary material, table S2. Thus, for each province i, we produced parameter estimates for the transmission rate, $\beta_{i}$ and ascertainment rate, $\alpha_{i}$, for each variant wave period. The procedure for parameter estimation using the EAKF is summarized in electronic supplementary material, algorithm S1.

The model is reinitialized for each wave and does not explicitly represent reinfections. In Mozambique, for the Delta and Omicron waves, the final $S_{i}$ estimated range of the prior wave (Alpha and Delta, respectively) was used as the susceptible initial prior, e.g. the *final* $S_{i}$ range of the Alpha wave =*initial prior* $S_{i}$ range of Delta wave (see electronic supplementary material, table S3). In Zimbabwe, because data were only available for the Omicron (BA.1) wave, *initial prior* $S_{i}$ was set to that of Mozambique for the same outbreak, as both countries are in the same region and experienced similar epidemic patterns.

The depletion of susceptibles, $S_{i}$, at the end of an outbreak reflected an approximation of the total new infections (both reported and unreported), given ongoing filter adjustment. The process of calculating cumulative infections for each wave highlights the direct relationship between the loss of susceptibles and the total number of infections. As the number of susceptibles decreases with each wave, the cumulative number of infections increases. The cumulative number of infections at any point in time is the sum of infections from all preceding waves.

To distinguish between different waves of infection, let w(w=1,2,3) denote the wave number. At the end of the wth wave, the number of susceptibles, $S_{iw}$, will decrease due to the infections that occurred during that wave. Consequently, to estimate the cumulative disease burden from the beginning of the pandemic across subsequent waves, the relationship between the depletion of susceptibles at the end of the wth wave and the cumulative number of infections can be expressed as


$$\begin{array}{lrlr} & \Delta S_{iw}\approx & 1-\frac{S_{iw}}{N_{i}} & \\ & \approx & C_{iw} & \end{array}$$

where $\Delta S_{iw}$ is the decrease in susceptibles during the *w*th wave, and $C_{iw}$ denotes the cumulative number of infections during that wave. The total cumulative number of infections at the end of the *w*th wave, $C_{iwTot}$ is the sum of the cumulative infections:


(2.1) $$\begin{array}{lrlr} & C_{iwtTot}\approx & \sum_{w=1}^{3}C_{iw} & \end{array}$$

Equation (2.1) provides an understanding of the full impact of the epidemic’s burden across multiple waves by quantifying the total cumulative infections, which include both reported and unreported cases.

### 2.2. The SEIR–EAKF framework

The EAKF assimilation algorithm has been used in previous studies of infectious disease dynamics to assimilate observations and conduct inference [31–33]. When coupled with the dynamic model, electronic supplementary material, equations (S1)–(S4), the combined SEIR–EAKF system iteratively optimizes the distribution of model state variables and parameters whenever new observations become available [34].

In general, sequential ensemble filtering is the problem of estimating the probability of the system state at a given time ${\text{x}}_{t}$ conditional on observations $O_{t}$ taken up until and inclusive of time *t*. After model initialization, the system integrates an ensemble of simulations forward in time to compute the prior distribution for the model state variables, parameters and the model-simulated case data. At the time of observation, the system is halted, and the EAKF is used to update the state variables and parameters based on those model-generated prior estimates and case data. The EAKF algorithm is applied with prescribed observational error variance (OEV), see §2.2.3. In this fashion, the ensemble simulations of the observed state variables (incidence) are updated to align with observations (see [34] for algorithmic details). The updates (posteriors) are determined by computing the Kalman gain using the latest observation and the distribution of current model states (the prior). The unobserved state variables and model parameters are then adjusted by the EAKF using cross-ensemble co-variability. The process is then repeated, with the posterior integrated into the next observation. Through this iterative optimization process, the ensemble of model simulations is better aligned to simulate current outbreak dynamics and estimate key parameters.

Here, a 300-member ensemble of simulations using the SEIR model, electronic supplementary material, equations (S1)–(S4), was coupled with the EAKF and case data. The state vector at time *t* is: ${\text{x}}_{t}=({\text{s}}_{t},\theta_{t})$, where ${\text{s}}_{t}=[S_{i},E_{i},I_{i}^{r},I_{i}^{u}]$ is the vector of the local state variables at time *t* and $\theta_{t}=[\mu ,Z,D,\beta_{i},\alpha_{i}]$ is the vector of model parameters. Using observation $O_{t}^{i}$ at time *t*, the posterior distribution of the system state is derived by applying Bayes’ rule (posterior ∝ prior × likelihood) to incorporate the new information. The EAKF deterministically adjusts the prior distribution to a posterior using Bayes rule while assuming a Gaussian distribution for both the prior and likelihood, which allows estimation of the first two moments (mean and covariance), leaving the higher-order moments unchanged. Bayes’ rule provides a target for updating the system state given an observation:


$$\begin{array}{r} p({\text{x}}_{t}|O_{1:t}^{i})\propto p({\text{x}}_{t}|O_{1:t-1}^{i})p(O_{1:t}^{i}|{\text{x}}_{t}). \end{array}$$

We used the daily number of newly reported cases in province i on a given day t, $O_{t}^{i}$, as observations. Unobserved variables, such as the susceptible population $S_{i}$, and model parameters, were adjusted in accordance with covariant relationships with the observed variable, i.e. reported new infections, $\left(\alpha_{i}E_{i}/Z\right)$ that arise naturally as a result of the dynamics of the system (see [34] for algorithmic details).

#### 2.2.1. State variables and parameter updates

State variables and the inferred model parameters ($\beta_{i}$ and $\alpha_{i}$) were updated for each province. Our parameters were updated locally, unlike in [8], where the parameters were updated globally. Estimating the parameters locally was motivated by the discrepancies found between population size and total incidence: Nampula province in Mozambique has the largest population, whereas Maputo Cidade (which is the capital city) has the smallest population. The reported cases indicated that Maputo Cidade had the highest number of COVID-19 cases, whereas Nampula had the fewest cases. This discrepancy may be due to the limited health care accessibility in Nampula compared with Maputo Cidade, which has the highest level of health care access among Mozambique provinces [35]. Such differences underpinned an expectation of different contact and ascertainment rates and motivated deriving local $\beta_{i}$ and $\alpha_{i}$ estimates for both countries.

The EAKF prediction–update cycle is performed sequentially, and an update is triggered by the arrival of new data (daily in this study) [34]. For the observable state variables, the ith ensemble member is updated by electronic supplementary material, equation (S6), while, for the unobserved variables/parameters $x^{i}$, the ith ensemble member is updated by electronic supplementary material, equation (S7).

#### 2.2.2. Initial priors

The initial 300-member ensemble of state variables and parameters was randomly drawn from a uniform distribution using Latin hypercube sampling and the initial range of values presented in electronic supplementary material, table S3; other parameters, D, Z and μ, were fixed. During filtering, ensemble members could move outside initial ranges; in such instances, the individual ensemble member would be resampled from the prior range.

The ascertainment rate initial prior range is $\alpha_{i}=\left[0.004,0.1\right]$ (which is low, corresponding to a maximum of one reported case for every ten infected) and was motivated by the study of Han *et al*. [10] which estimated a mean ascertainment rate of α=0.057 among all African countries (including Mozambique and Zimbabwe), with the lowest α=0.0002 in Sao Tomé and Príncipe and the highest α=0.3 in Libya. In the Southern Africa region, where Mozambique and Zimbabwe are situated, Han *et al*. [10] found the highest mean ascertainment rate to be α=0.127 in South Africa, whereas Mozambique and Zimbabwe had α<0.1. The low estimates for both countries motivated the selection of a narrower initial prior range for the ascertainment rates. Furthermore, the study by Evans *et al*. [36] highlighted that the lower-than-expected case burdens in Madagascar were explained solely by detection rates from 0.1% to 1% (or α=0.001 to α=0.01). On the other hand, due to the heterogeneity of reported cases across provinces in Mozambique, the initial prior lower bound for $\alpha_{i}$ was varied by province for both countries (see electronic supplementary material, table S3). For province i, we applied the formula: lower bound $\alpha_{i}=(\text{total reported cases})/0.5N_{i}$, assuming 50% of the population was infected at the end of the three variant waves (Alpha, Delta and Omicron (BA.1)). An exception was Maputo Cidade, which had a different initial prior range $\alpha_{i}=\left[0.14,0.2\right]$, because Maputo Cidade is the capital city of Mozambique; it has more resources, including a greater availability of healthcare and improved reporting of COVID cases. This leads to an expected higher reporting rate. Again, although Maputo Cidade has the lowest population, it is urban with high population density, the most advanced healthcare centres in the country, and thus the highest reported number of cases. Generally, the remaining Mozambique provinces had ascertainment rate lower bounds ranging from $\alpha_{i}=0,004$ to $\alpha_{i}=0.03$, whereas for Zimbabwe, lower bounds ranged from $\alpha_{i}=0.01$ to $\alpha_{i}=0.04$. The $S_{i}$ initial priors varied for each wave because the model was run piecewise for the periods as described above and in electronic supplementary material, table S3.

#### 2.2.3. Observational error variance

Using the EAKF requires specification of error for both the simulated model output and observations. The model error may be easily computed as the variance of the 300-member ensemble. For each $O_{t}^{i}$, we specify the OEV in province *i* at time *t* as


(2.2) $$\begin{array}{lrr} & OEV_{t}^{i}=max(4,\frac{(O_{t}^{i}{)}^{2}}{4}). & \end{array}$$

An OEV of this form has been used successfully for inference and forecasting for infectious diseases, such as influenza [32,37] and COVID-19 [8].

#### 2.2.4. Filter divergence

As successive observations are assimilated, there is a tendency for the variance between the ensemble members to decrease due to repeated filter adjustments. This may potentially lead to filter divergence, in which the ensemble error variance is so minimal that the fitting process essentially ignores the observations [34]. To prevent filter divergence, the prior ensemble was inflated by a multiplicative factor λ=0.015, before each daily assimilation and calculation of the posterior (see electronic supplementary material, equation (S8)). The inflation was applied to all state variables and estimated parameters, i.e. $\beta_{i}$ (transmission rate) and $\alpha_{i}$ (ascertainment rate).

### 2.3. Synthetic testing

To validate EAKF inference with the SEIR model, we generated synthetic model-simulated COVID-19 two consecutive outbreaks (defined as the ‘truth’), defined by model electronic supplementary material, equations (S1)–(S4). We tested the ability of the model/filter framework to identify the true model state variables and parameters at the end of each outbreak. Two sets of truth, each with two consecutive waves, were generated: synthetic outbreak 1 with Wave 1(a) and Wave 2(a) (see electronic supplementary material, figure S2) and synthetic outbreak 2 (with Wave 1(b) and Wave 2(b)). $S_{i}$ initial prior was the same as the Alpha and Delta waves (see electronic supplementary material, table S3) for the consecutive waves, respectively. Synthetic observations of new daily reported cases (reported new infections, $(\alpha_{i}E_{i}/Z)$) were then generated by adding normally distributed random observational error calculated as described in equation (2.2). The resulting time series of synthetic error-laden observational records was smoothed using a 7-day moving average and then used for assimilation in the combined SEIR–EAKF framework to determine whether the system can reliably identify known parameter values.

### 2.4. Parameter estimation using real data

The SEIR–EAKF framework was applied in isolation to the 11 Mozambique provinces and, separately, the 10 Zimbabwe provinces, as illustrated in electronic supplementary material, figure S1. The Mozambique COVID-19 data were transformed from weekly cases to daily cases using a linear interpolation method, and piecewise parameter estimation was carried out for the model’s local parameters, $\beta_{i}$ and $\alpha_{i}$, for each variant wave as described in electronic supplementary material, table S2 and §S1.4.

## 3. Results

### 3.1. Data description

**Figure 2.**
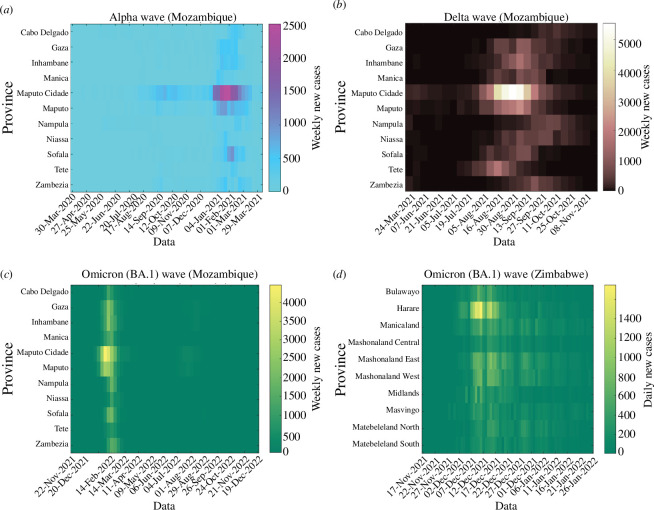
Heat maps of daily and weekly provincial-level COVID-19 infections in Mozambique and Zimbabwe between March 2020 and December 2022, based on data released by the Mozambique Ministry of Health-SIGILIA and DISA [29] and the Zimbabwe COVID-19 hub [30].

The reported daily COVID-19 cases used for this study span three variant waves (Alpha, Delta and Omicron (BA.1)) during the first 3 years of the pandemic, i.e. from 30th March 2020 to 26th December 2022 for Mozambique and from 18th November 2021 to 26th January 2022 for Zimbabwe. The available Zimbabwe provincial data began in the middle of the Delta variant wave; therefore, we only utilized the data covering the Omicron (BA.1) wave. At the provincial scale, COVID-19 daily cases were obtained from the Zimbabwe COVID-19 hub [30], and weekly cases for Mozambique were obtained from the Mozambique Ministry of Health-SIGILIA and DISA [29]. Heat maps of daily and weekly counts of COVID-19 cases, for each variant period, in each province of both countries are presented in figure 2, while plots of the disaggregated time series of COVID-19 infections for the provinces are reported in electronic supplementary material, figure S1. Provincial population data were taken from the Zimbabwe National Statistics Agency [38] and Instituto Nacional de Estatistica Moçambique [39] for Zimbabwe and Mozambique, respectively.

### 3.2. Validation of the model-inference system using synthetic outbreaks

The synthetic observations of COVID-19 cases, along with their defined OEV, were used to test whether the SEIR–EAKF system could estimate unobserved state variables and parameters ($\beta_{i}$ and $\alpha_{i}$) accurately. Electronic supplementary material, figures S3–S10 depict the parameter estimates for the simulation period; the posterior parameter estimates ($\beta_{i}$ and $\alpha_{i}$) converged near their target values, and the reproductive number at the end of each variant wave, $Rt_{i}$, was correctly estimated. The ensemble posterior distributions at the end of each outbreak were well constrained for all the generated outbreaks. These synthetic tests reveal that the model-inference system is able to estimate system parameters over two successive waves.

### 3.3. Mozambique parameter estimates

The estimates for $\beta_{i}$ and $\alpha_{i}$ at the end of each variant outbreak in Mozambique, along with their associated 95% credible intervals (CrIs), are presented in table 1. With the exception of Maputo Cidade, for all provinces, the mean ascertainment rates estimates across all three waves ranged from $\alpha_{i}=0.08$ to $\alpha_{i}=0.09$. For the Delta wave, Nampula exhibited the lowest rate of $\alpha_{7}=0.03$. On the other hand, Maputo Cidade, with its unique prior range (see electronic supplementary material, §S1.4), had an estimate around $\alpha_{5}=0.17$ across all variant waves. The local $\beta_{i}$ mean estimates and, by extension, $Rt_{i}$ increased with each successive wave: for the Alpha wave, the mean estimates ranged from $\beta_{4}=0.55$$\left(Rt_{4}=1.41\right)$ for Manica to $\beta_{5}=0.67$$\left(Rt_{5}=1.78\right)$ for Maputo Cidade; the estimates were slightly higher for the Delta wave, ranging from $\beta_{9}=0.57$$\left(Rt_{9}=1.44\right)$ for Sofala to $\beta_{2}=1.49$$\left(Rt_{2}=3.70\right)$ for Gaza; and the highest estimates were for the Omicron (BA.1) wave ranging from $\beta_{7}=1.12$$\left(Rt_{7}=2.78\right)$ for Nampula to $\beta_{3}=1.70$$\left(Rt_{3}=4.31\right)$ for Inhambane.

**Table 1.** EAKF estimated parameters and credible intervals at the end of each Mozambique variant period.

province	parameter	prior	EAKF estimate (Alpha)		EAKF estimate (Delta)		EAKF estimate (Omicron (BA.1))	
			mean	95% CrI	mean	95% CrI	mean	95% CrI
transmission rates								
Cabo Delgado	$\beta_{1}$	[0.5, 2.0]	0.6038	(0.5032, 0.8037)	1.1571	(0.7873, 1.6191)	1.1528	(1.0684, 1.4876)
Gaza	$\beta_{2}$		0.5643	(0.5036, 0.7021)	1.4886	(0.6507, 1.9467)	1.5630	(0.6812, 1.9322)
Inhambane	$\beta_{3}$		0.5830	(0.5040, 0.7898)	1.2970	(0.6593, 1.9414)	1.6998	(0.9363, 1.9593)
Manica	$\beta_{4}$		0.5526	(0.5021, 0.6768)	0.6362	(0.5073, 1.3342)	1.4411	(0.6655, 1.9614)
Maputo Cidade	$\beta_{5}$		0.6681	(0.5157, 0.9132)	1.1154	(0.5938, 1.9074)	1.5664	(0.7519, 1.9561)
Maputo	$\beta_{6}$		0.6136	(0.5135, 0.8306)	1.3520	(0.6281, 1.9099)	1.5299	(0.8293, 1.9683)
Nampula	$\beta_{7}$		0.5645	(0.5035, 0.6679)	0.9902	(0.6117, 1.7501)	1.1174	(0.5324, 1.9316)
Niassa	$\beta_{8}$		0.6059	(0.5026, 0.7684)	0.7763	(0.5156, 1.5475)	1.5890	(0.7134, 1.9788)
Sofala	$\beta_{9}$		0.6060	(0.5046, 0.8032)	0.5680	(0.5035, 0.9613)	1.3762	(0.7603, 1.9652)
Tete	$\beta_{10}$		0.6082	(0.5036, 0.8303)	0.6315	(0.5107, 1.1309)	1.1819	(0.5908, 1.9180)
Zambezia	$\beta_{11}$		0.5796	(0.5055, 0.7656)	0.8914	(0.5229, 1.7148)	1.3361	(0.6657, 1.9511)
ascertainment rates								
Cabo Delgado	$\alpha_{1}$	[0.007, 0.1]	0.0923	(0.0744, 0.0993)	0.0833	(0.0158, 0.0990)	0.0798	(0.0301, 0.0983)
Gaza	$\alpha_{2}$	[0.02, 0.1]	0.0883	(0.0606, 0.0998)	0.0622	(0.0264, 0.0978)	0.0718	(0.0316, 0.0991)
Inhambane	$\alpha_{3}$	[0.03, 0.1]	0.0904	(0.0554, 0.0998)	0.0627	(0.0330, 0.0965)	0.0881	(0.0570, 0.0991)
Manica	$\alpha_{4}$	[0.01, 0.1]	0.0922	(0.0739, 0.0996)	0.0881	(0.0426, 0.0987)	0.0836	(0.0203, 0.0994)
Maputo Cidade	$\alpha_{5}$	[0.14, 0.2]	0.1739	(0.1419, 0.1988)	0.1760	(0.1477, 0.1990)	0.1729	(0.1411, 0.1977)
Maputo	$\alpha_{6}$	[0.03, 0.1]	0.0836	(0.0558, 0.0985)	0.0640	(0.0334, 0.0986)	0.0677	(0.0379, 0.0970)
Nampula	$\alpha_{7}$	[0.004, 0.1]	0.0911	(0.0658, 0.0995)	0.0318	(0.0174, 0.0754)	0.0867	(0.0163, 0.0987)
Niassa	$\alpha_{8}$	[0.01, 0.1]	0.0930	(0.0684, 0.0998)	0.0812	(0.0371, 0.0992)	0.0888	(0.0403, 0.0979)
Sofala	$\alpha_{9}$	[0.01, 0.1]	0.0872	(0.0519, 0.0997)	0.0849	(0.0455, 0.0995)	0.0827	(0.0176, 0.0998)
Tete	$\alpha_{10}$	[0.009, 0.1]	0.0902	(0.0684, 0.0995)	0.0862	(0.0468, 0.0996)	0.0787	(0.0468, 0.0996)
Zambezia	$\alpha_{11}$	[0.005, 0.1]	0.0902	(0.0543, 0.0996)	0.0884	(0.0320, 0.0990)	0.0879	(0.0513, 0.0996)
reproductive number								
Cabo Delgado	$R\,t_{1}$		1.5367	(1.2760, 2.0486)	2.9193	(1.9882, 4.0874)	2.9056	(1.3940, 4.8801)
Gaza	$R\,t_{2}$		1.4317	(1.2738, 1.7767)	3.6994	(1.6392, 4.8517)	3.9199	(1.7308, 4.8177)
Inhambane	$R\,t_{3}$		1.4748	(1.2827, 2.0140)	3.2424	(1.6336, 4.8583)	4.3049	(2.3873, 4.9700)
Manica	$R\,t_{4}$		1.4100	(1.2765, 1.7275)	1.6184	(1.2840, 3.3278)	3.6355	(1.6712, 4.9141)
Maputo Cidade	$R\,t_{5}$		1.7843	(1.3755, 2.4536)	2.9981	(1.5922, 5.1157)	4.1683	(1.9782, 5.2456)
Maputo	$R\,t_{6}$		1.5550	(1.3017, 2.0922)	3.3779	(1.5619, 4.8312)	3.8328	(2.1190, 4.9489)
Nampula	$R\,t_{7}$		1.4404	(1.2798 1.7002)	2.4349	(1.5105, 4.2809)	2.7798	(1.3296, 4.8345)
Niassa	$R\,t_{8}$		1.5362	(1.2773, 1.9420)	1.9631	(1.3020, 3.8910)	4.0304	(1.8178, 4.9980)
Sofala	$R\,t_{9}$		1.5218	(1.2829, 2.0447)	1.4373	(1.2688, 2.4461)	3.4886	(1.9383, 4.9815)
Tete	$R\,t_{10}$		1.5395	(1.2799, 2.1200)	1.5965	(1.2932, 2.8883)	2.9824	(1.5107, 4.8073)
Zambezia	$R\,t_{11}$		1.4710	(1.2825, 1.9245)	2.2548	(1.3290, 4.3444)	3.4098	(1.6867, 4.9622)

Electronic supplementary material, figures S11–S13 illustrate the time evolution of the parameter posteriors (means and 95% CrIs) for the Alpha, Delta and Omicron (BA.1) variant waves, respectively, plotted against their prior range. The initial drift in the first month is likely a reflection of the time needed for the EAKF to learn the system [34]. The $\alpha_{i}$ estimates at the end of the outbreaks were closer to the upper bound of 0.2 for Maputo Cidade and 0.1 for the rest of the provinces. For the Alpha and Delta waves, $\beta_{i}$ converges to a solution during January 2021 and August 2021, respectively, as cases rise; on the other hand, for the Omicron (BA.1) wave, $\beta_{i}$ convergence to a solution is less clear, with a broader posterior and higher mean estimate.

The posterior fits of new reported cases to province-specific actual data [29] were generally good for all provinces in Mozambique for all variant waves, as demonstrated in electronic supplementary material, figures S14–S16. The model-inference framework was able to reproduce even complicated outbreak structures characterized by multiple peaks, such as during the Alpha wave in Nampula, Tete and Zambezia.

**Figure 3.**
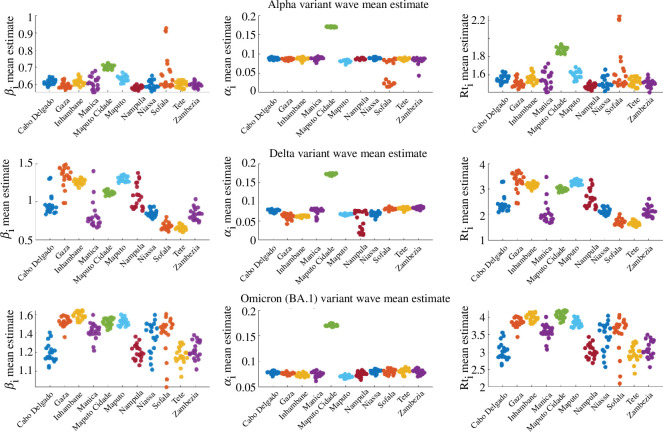
Distribution of mean parameter estimates ($\alpha_{i}$ and $\beta_{i}$, and the corresponding $Rt_{i}$) for the 11 Mozambique provinces at the end of variant waves (Alpha, Delta and Omicron (BA.1)) for 20 runs.

In order to check the consistency of the parameter estimates for each variant wave, presented in table 1, SEIR–EAKF system simulations were repeated 20 times. The mean parameter estimates ($\beta_{i}$ and $\alpha_{i}$) and the corresponding mean $Rt_{i}$ for the runs are illustrated in figure 3; they showed consistency across the variant waves. Electronic supplementary material, figures S20–S22 show the joint distributions of $\beta_{i}$ and $\alpha_{i}$ for the variant waves in Mozambique for the 20 runs (shown in figure 3). Electronic supplementary material, figure S19a–c shows the posterior mean estimates of susceptibility % at the end of each variant wave. An estimated 75% to 95% of the population remained susceptible at the end of the Alpha wave (in March 2021), furthermore, from 15% to 50% and from 5% to 30% remained susceptible after the Delta (in October 2021) and Omicron (BA.1) (in March 2022) waves, respectively.

To quantify the burden of COVID-19 infections at the end of each variant period, we estimated the total cumulative infections, i.e. both reported and unreported infections, by applying equation (2.1). This process was repeated for the three waves of infection. At the end of the Alpha wave, we calculated the total cumulative infections. The loss of susceptibles during this wave directly contributed to the cumulative number of infections. For the Delta wave, we again applied equation (2.1) to estimate the total cumulative infections, which included infections from both the Alpha and Delta waves. The further reduction in susceptibles was reflected in the increased cumulative infection count, which now included infections from both waves. Similarly, during the Omicron wave, the same method was used to estimate the cumulative infections, encompassing the sum of all three waves. The continued loss of susceptibles during the Omicron wave resulted in an even higher cumulative infection count.

**Figure 4.**
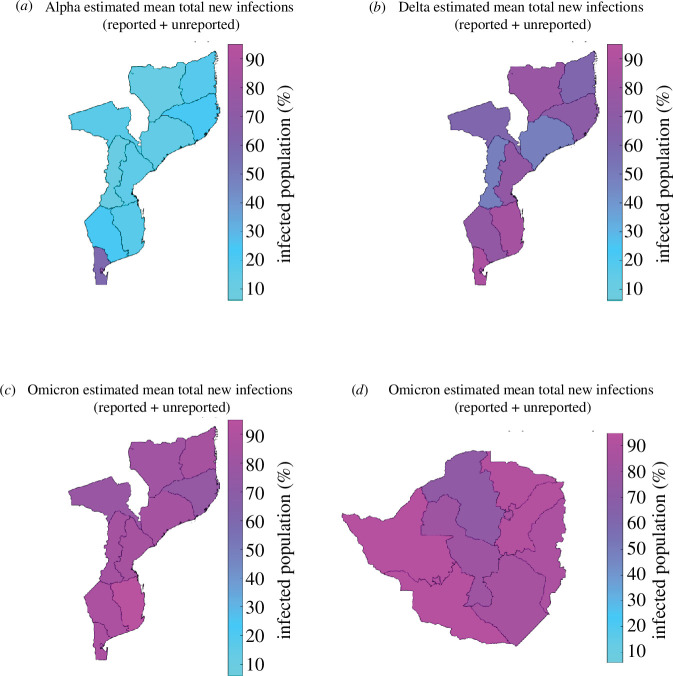
Spatial distribution of estimated SARS-CoV-2 infections (both reported and unreported) per VOC for Mozambique and Zimbabwe provinces between March 2020 and December 2022 [29,30]. Colours towards red indicate more infections. (*a*) Alpha estimated mean total new infections (reported + unreported), (*b*) Delta estimated mean total new infections (reported + unreported), (*c*) Omicron estimated mean total new infections (reported + unreported) and (*d*) Omicron estimated mean total new infections (reported + unreported).

Figure 4*a–c* displays the spatial distribution of the estimated total new infections (reported and unreported) across all Mozambique provinces. We estimated an increase in the proportion of the infected population across the three VOC (Alpha, Delta and Omicron (BA.1)): the Alpha wave estimates were below 20% for the majority of the country; in contrast, Maputo Cidade and Maputo showed the highest burdens of 72% and 57%, respectively. The cumulative infection estimates increased from 6% to 72% (Alpha wave), from 46% to 88% (Delta wave) and from 74% to 95% (Omicron BA.1 wave).

### 3.4. Zimbabwe parameter estimates

Table 2 presents the parameter estimates ($\beta_{i}$ and $\alpha_{i}$) and associated 95% CrIs at the end of the Omicron (BA.1) variant outbreak. The estimates for the ascertainment rates ranged from $\alpha_{i}=0.08$ to $\alpha_{i}=0.09$, which is in line with Mozambique estimates (shown in table 1). On the other hand, the mean estimates for the transmission rate ranged from $\beta_{3}=1.13$ for Manicaland to $\beta_{9}=1.66$ for Matebeleland North, with reproductive numbers from $Rt_{3}=3.69$ to $Rt_{9}=5.41$, respectively. The time evolution of $\alpha_{i}$ and $\beta_{i}$ parameter posteriors (means and 95% CrI) over the course of the Omicron (BA.1) wave, plotted against their prior range, is presented in electronic supplementary material, figure S17. The $\alpha_{i}$ estimates drifted higher towards the upper bound (0.1) during December 2021, a level maintained until the end of the outbreak. The convergence of $\beta_{i}$ to a solution is less clear, with a broader posterior and higher mean estimate.

**Table 2.** EAKF estimated parameters and credible intervals for the Zimbabwe Omicron (BA.1) variant period.

province	parameter	prior	EAKF estimate (Omicron (BA.1))	
			mean	95% CrI
transmission rates				
Bulawayo	$\beta_{1}$	[0.5, 2.0]	1.6529	(1.0081, 1.9813)
Harare	$\beta_{2}$		1.6407	(1.0550, 1.9762)
Manicaland	$\beta_{3}$		1.1327	(0.5971, 1.9040)
Mashonaland Central	$\beta_{4}$		1.2506	(0.7189, 1.9148)
Mashonaland East	$\beta_{5}$		1.5438	(0.8128, 1.9457)
Mashonaland West	$\beta_{6}$		1.4040	(0.8200, 1.9323)
Midlands	$\beta_{7}$		1.3669	(0.6988, 1.9702)
Masvingo	$\beta_{8}$		1.5457	(0.8012, 1.9652)
Matabeleland North	$\beta_{9}$		1.6567	(1.0512, 1.9716)
Matabeleland South	$\beta_{10}$		1.6446	(0.9344, 1.9848)
ascertainment rates				
Bulawayo	$\alpha_{1}$	[0.04, 0.1]	0.0911	(0.0668, 0.0997)
Harare	$\alpha_{2}$	[0.03, 0.1]	0.0924	(0.0744, 0.0997)
Manicaland	$\alpha_{3}$	[0.02, 0.1]	0.0864	(0.0387, 0.0990)
Mashonaland Central	$\alpha_{4}$	[0.01, 0.1]	0.0929	(0.0589, 0.0997)
Mashonaland East	$\alpha_{5}$	[0.03, 0.1]	0.0871	(0.0562, 0.0984)
Mashonaland West	$\alpha_{6}$	[0.02, 0.1]	0.0930	(0.0694, 0.0997)
Midlands	$\alpha_{7}$	[0.01, 0.1]	0.0917	(0.0534, 0.0993)
Masvingo	$\alpha_{8}$	[0.02, 0.1]	0.0876	(0.0474, 0.0989)
Matabeleland North	$\alpha_{9}$	[0.04, 0.1]	0.0857	(0.0499, 0.0992)
Matabeleland South	$\alpha_{10}$	[0.03, 0.1]	0.0846	(0.0489, 0.0991)
reproductive number				
Bulawayo	$Rt_{1}$		5.4010	(3.3035, 6.4817)
Harare	$Rt_{2}$		5.3709	(3.4402, 6.4669)
Manicaland	$Rt_{3}$		3.6866	(1.9242, 6.2350)
Mashonaland Central	$Rt_{4}$		4.0797	(2.3522, 6.2600)
Mashonaland East	$Rt_{5}$		5.0435	(2.6502, 6.3627)
Mashonaland West	$Rt_{6}$		4.6015	(2.6877, 6.3179)
Midlands	$Rt_{7}$		4.4749	(2.2919, 6.4159)
Masvingo	$Rt_{8}$		5.0560	(2.0240, 3.0989)
Matabeleland North	$Rt_{9}$		5.4064	(3.4283, 6.4578)
Matabeleland South	$Rt_{10}$		5.3699	(3.0497, 6.4785)

**Figure 5.**
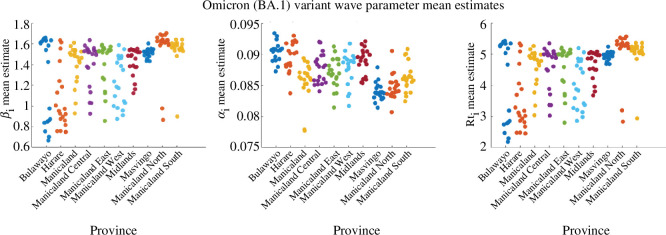
Distribution of mean parameter estimates ($\alpha_{i}$, $\beta_{i}$ and corresponding $Rt_{i}$) for the 10 Zimbabwe provinces at the end of Omicron (BA.1) wave for 20 runs.

Electronic supplementary material, figure S18 demonstrates a good model fit to provincial data [30] for the outbreak, with the posterior estimates capturing the outbreak peaks for all 10 provinces. The consistency of the parameter estimates (table 2) was evaluated by running the model 20 times, and the distribution of the mean parameter estimates ($\beta_{i}$ and $\alpha_{i}$) at the end of the outbreak and the corresponding mean $Rt_{i}$ for the runs are shown in figure 5. The parameter distribution indicates that the mean estimates were consistent with the Omicron (BA.1) wave estimates for Mozambique. Electronic supplementary material, figure S23 shows the joint distribution for $\beta_{i}$ and $\alpha_{i}$ for the 20 runs (shown in figure 5), where the probability density is centred around the estimates in table 2. Electronic supplementary material, figure S19d shows the posterior mean estimates of susceptibility % at the end of the Omicron (BA.1) wave (in January 2022), where from 15% to 40% of the population remained susceptible at the end of that outbreak.

The spatial distribution of the estimated mean total new COVID-19 infections (both reported and unreported) at the end of the Omicron (BA.1) wave is summarized in figure 4*d*. The findings indicate that the infected population at the end of the outbreak ranged from 71% to 93%.

## 4. Discussion

Although the emergence of COVID-19 and its rapid spread created a public health emergency of international concern, the impact of the pandemic in Sub-Saharan Africa, as documented in cases, hospitalizations and deaths, appears far lower than in the Americas, Europe and Asia [40]. We utilized a model-inference framework to shed light on disease dynamics in two southern African countries, i.e. Mozambique and Zimbabwe, during the first 3 years of the pandemic. While accounting for undocumented cases, we estimated key epidemiological parameters, namely the transmission rate and the ascertainment rate at the end of each outbreak. Our study provides several insights into the disease burden of infections (reported and unreported) for three VOCs, i.e. Alpha, Delta and Omicron (BA.1). While we focus on the Mozambique and Zimbabwe cases, our framework can be applied to other African countries given the low documented impact of the disease in this region.

The ascertainment rate, α, estimates are very low for Mozambique and Zimbabwe provinces (ranging from α=0.08 to α=0.09). Our estimates in both countries are in line with the studies by Han *et al*. [10] and Evans *et al*. [36], where similar transmission dynamics, that is, low ascertainment rates, were experienced by the two southern African countries. The mean estimated ascertainment rates for all 54 African countries were 5.37% (α=0.0537) [10]. Mozambique and Zimbabwe experienced similar epidemiological dynamics for the Omicron (BA.1) wave with low ascertainment rates compared to elsewhere. Within the same region, South Africa had the highest estimated ascertainment rate of 12.7% (α=0.127). For the other regions, China had an estimated rate of α=0.14 at the beginning of the pandemic [8], during 2020, the USA overall ascertainment rate, α, was approximately 0.22 [17], and the European Union had rates close to 0.25 in January 2022 [41]. The lower ascertainment rates in Africa indicate that the majority of infections were never documented as cases; by extension, many severe cases requiring hospitalization or resulting in death may have been missed due to limited access to health care and testing facilities. The estimated low ascertainment rate highlights the need for enhanced reporting and surveillance mechanisms in Sub-Saharan Africa. Mozambique experienced natural disasters and conflict, which hampered access to health care facilities. Prior to the pandemic, the country was devastated by two consecutive deadly and destructive cyclones (Idai and Kenneth), which caused massive destruction to infrastructure; consequently, accessibility in some places became more difficult. Furthermore, conflict in northern Mozambique produced additional challenges during the pandemic by displacing many people, hence making it difficult to control the disease [42].

The transmission rate estimates, and by extension the reproductive number, rose with each successive wave. This matches observations in the Americas, Africa, Europe and Asia that the Omicron variant, in particular, was more transmissible than the preceding Delta variant [43,44], which in turn had greater transmissibility than the preceding Alpha variant [45,46]. The variability for some province-parameter combinations indicates uncertainty in the mean estimate. Mozambique and Zimbabwe deployed public health and social measures to mitigate the spread of COVID-19. These included travel restrictions, lockdowns, social distancing measures, compulsory mask wearing, contact tracing and testing, school closures and use of personal protective equipment among health workers [47–50].

Our model was fitted to provincially reported cases. We find that representing and accounting for unreported cases across provinces is crucial for estimating the true disease burden, i.e. new infections (reported and unreported), at the end of each variant wave. The low numbers of confirmed cases in Africa were generally a poor indicator of the true incidence of infection, which may be attributed to limited testing capacity and health system access. Overall, the estimated disease burden in Mozambique and Zimbabwe was much higher than the documented reported cases for each VOC (see [29,30], electronic supplementary material, tables S4 and S5). The estimates highlight that by the end of the Omicron (BA.1) wave, almost the entire population had been infected, which is in agreement with the estimates of 93% cumulative total infection after the same wave in South Africa [16]. Our results indicate that in African countries, efforts must be made to improve the timely reporting and surveillance of public health threats.

There are limitations to our model. First, even though human mobility plays a crucial role in the transmission of SARS-CoV-2, the unavailability of interprovincial human movement data for both Mozambique and Zimbabwe was a drawback; hence, we implemented the metapopulation model without movement between provinces. Second, the model does not explicitly account for reinfections, but the filter adjusts the number of susceptibles, thereby implicitly accounting for increasing susceptibility due to immune escape. Neither births nor deaths are accounted for—the assumption is a population at steady state (births replacing deaths over the study period). Finally, the models considered here do not explicitly account for preventative measures; however, note that the inference conducted estimates the transmission rate, β, in the presence of these controls, as reflected in the observations. That is, any suppression or relaxation of controls that influence contact and transmission rates would be estimated as changes to β and $R_{t}$ over time.

In conclusion, we developed an inference-based transmission model to aid in understanding the evolving dynamics of SARS-CoV-2 in Mozambique and Zimbabwe. By taking unreported cases into account, we estimated key epidemiologic characteristics, i.e. the transmission rate and ascertainment rate. This approach and findings are relevant for countries with less comprehensive surveillance systems. The findings of this study on the disease burden can help guide future public health planning. In particular, they shed light on respiratory virus transmission dynamics in two African countries that have been little investigated to date.

## Data Availability

Data and relevant code for this research work are stored in GitHub: https://github.com/roseshava/SEIR-EAKF_Moza_Zim [51] and have been archived within the Zenodo repository: https://doi.org/10.5281/zenodo.13992818 [52]. The data that supports the findings of this study are available upon request from INS Mozambique Sérgio Chicumbe -Directorate for health research, mailto:sergio.chicumbe@ins.gov.mz. Supplementary material is available online [53].
